# Supplementary material for: The effectiveness of digital delivery versus group-based face-to-face delivery of the English National Health Service Type 2 Diabetes Prevention Programme: a non-inferiority retrospective cohort comparison study
Source: BMC Health Serv Res. 2023 Dec 18;23:1434. doi: 10.1186/s12913-023-10365-2 (PMC10729322; doi:10.1186/s12913-023-10365-2)
Supplement: Supplementary file 1 — Additional file 1. [file 12913_2023_10365_MOESM1_ESM.docx]

**The effectiveness of digital delivery versus group-based face-to-face delivery of the English National Health Service Type 2 Diabetes Prevention Programme: a non-inferiority retrospective cohort comparison study**

**Additional File 1**

**1. Sample size justification**

The original sample size calculation is shown in the online pre-registration on the Open Science Framework repository: <https://osf.io/a9pbw>. In the protocol, in error, we used the 12-month non-inferiority margin instead of the 6-month margin for the change in weight calculations. The revised sample size calculation is presented here.

In the pilot digital data, there are 1025 individuals from a digital-only area who have both a baseline and 6-month weight measure. The pooled standard deviation of change in weight from baseline to 6 months for eligible participants in the digital-only and face-to-face cohorts is 3.89kg. Assuming a 5/1 ratio of F2f participants to digital participants, to detect a non-inferiority margin of 1kg in a two-sample t-test with 90% power, a significance level of 0.05 and a population standard deviation of 3.89, 156 participants in the digital-only group are required.

**2. Summary of the timings of the outcome measures**

Table S1 Summary of the timings of the 6-month and 12-month weight measures in the face-to-face, digital-only and digital-choice groups

|  | **Face-to-face** | **Digital-only** | **Digital-choice** |
| --- | --- | --- | --- |
| Timing of 6-month weight measure  n  4 months  5 months  6 months  7 months  Median (IQR) | 32744  4212 (12.9%)  18263 (55.8%)  8802 (26.9%)  1467 (4.48%)  5 (5, 6) | 1025  107 (10.4%)  446 (43.5%)  345 (33.7%)  127 (12.4%)  5 (5, 6) | 830  34 (4.10%)  479 (57.7%)  254 (30.6%)  63 (7.59)  5 (5, 6) |
| Timing of 12-month weight measure  n  8 months  9 months  10 months  11 months  12 months  13 months  Median (IQR) | 23458  8480 (36.2%)  4981 (21.2%)  2878 (12.3%)  4322 (18.4%)  1977 (8.43%)  820 (3.50%)  9 (8, 11) | 818  7 (0.86%)  31 (3.79%)  84 (10.3%)  297 (36.3%)  261 (31.9%)  138 (16.9%)  11 (11, 12) | 765  21 (2.75%)  34 (4.44%)  51 (6.67%)  314 (41.1%)  268 (35.0%)  77 (10.1%)  11 (11, 12) |

**3. Summary of baseline characteristics in the matched cohorts**

Table S2 Baseline characteristics of participants in matching cohorts for the change in weight at 6m analyses comparing digital-only and digital-choice with face-to-face

|  | **Digital only vs face-to-face**  **Matching rate: 98.8%** | | **Digital choice vs face-to-face Matching rate: 99.2%** | |
| --- | --- | --- | --- | --- |
|  | **Digital only (n=962)** | **Face-to-face (n=4764)** | **Digital choice (n=758)** | **Face-to-face (n=3746)** |
| **Sex**  Male, n (%) Female, n (%) | 439 (45.6%)  523 (54.4%) | 2176 (45.7%)  2588 (54.3%) | 371 (48.9%)  387 (51.1%) | 1840 (49.1%)  1906 (50.9%) |
| **Age at referral**  Mean (SD) Median (IQR) | 60.0 (11.7)  61 (52, 69) | 60.8 (11.5)  62 (53, 70) | 60.0 (11.3)  61 (53, 69) | 61.0 (11.1)  62 (54, 69) |
| **Ethnicity***  White, n (%)  Mixed, n (%)  Asian, n (%)  Black, n (%)  Other, n (%) | 755 (78.5%)  12 (1.25%)  138 (14.4%)  55 (5.72%)  2 (0.21%) | 3760 (78.9%)  48 (1.01%)  678 (14.2%)  270 (5.67%)  8 (0.17%) | 640 (84.4%)  16 (2.11%)  75 (9.89%)  23 (3.03%)  4 (0.53%) | 3188 (85.1%)  62 (1.66%)  365 (9.74%)  115 (3.07%)  16 (0.43%) |
| **IMD Quintile**^†^  1 (Most deprived), n(%)  2, n(%)  3, n(%)  4, n(%)  5 (Least deprived), n(%) | 142 (14.8%)  214 (22.3%)  288 (29.9%)  196 (20.4%)  122 (12.7%) | 695 (14.6%)  1066 (22.4%)  1421 (29.8%)  978 (20.5%)  604 (12.7%) | 178 (23.5%)  101 (13.3%)  96 (12.7%)  175 (23.1%)  208 (27.4%) | 870 (23.2%)  498 (13.3%)  480 (12.8%)  866 (23.1%)  1032 (27.6%) |
| **Weight in kg at baseline**  Mean (SD) Median (IQR) | 87.6 (19.2)  85.0 (73.9, 99.0) | 85.9 (19.4)  83.6 (72.1, 96.4) | 86.9 (19.1)  84.8 (73.9, 98.0) | 87.2 (19.4)  85.4 (73.4, 98.2) |
| **BMI at baseline^a^**  Mean (SD) Median (IQR) | 31.1 (6.00)  30.1 (26.6, 34.5) | 30.7 (6.1)  29.8 (26.5, 33.8) | 30.5 (6.1)  29.3 (26.2, 33.4) | 30.9 (6.2)  30.0 (26.6, 34.1) |

* Asian’ comprises those reporting Indian, Pakistani, Bangladeshi, Chinese or ‘other Asian’ ethnicity; ‘Black’ comprises those reporting Caribbean, African or ‘other Black’ ethnicity; ‘Mixed’ comprises people with a Mixed ethnic background and ‘Other’ comprises those reporting any other ethnicity.
^†^‘IMD’ – Index of Multiple Deprivation (English, 2015)
^a^ ‘BMI’ – Body Mass Index

Table S3 Baseline characteristics of participants in matching cohorts for the change in weight at 12m analyses comparing digital-only and digital-choice with face-to-face

|  | **Digital only vs face-to-face**  **Matching rate: 99.4%** | | **Digital choice vs face-to-face**  **Matching rate: 98.1%** | |
| --- | --- | --- | --- | --- |
|  | **Digital only (n=770)** | **Face-to-face (n=3819)** | **Digital only (n=687)** | **Face-to-face (n=3353)** |
| **Sex**  Male, n(%) Female, n(%) | 366 (47.5%)  404 (52.5%) | 1809 (47.4%)  2010 (52.6%) | 334 (48.6%)  353 (51.4%) | 1633 (48.7%)  1720 (51.3%) |
| **Age at referral**  Mean (SD) Median (IQR) | 60.9 (11.0)  62 (53, 70) | 61.7 (11.0)  63 (54, 70) | 60.1 (11.1)  61 (53, 68) | 61.3 (10.9)  62 (54, 69) |
| **Ethnicity***  White, n(%)  Mixed, n(%)  Asian, n(%)  Black, n(%)  Other, n(%) | 617 (80.1%)  6 (0.78%)  103 (13.4%)  43 (5.58%)  1 (0.13%) | 3070 (80.4%)  28 (0.73%)  506 (13.3%)  213 (5.58%)  2 (0.05%) | 583 (84.9%)  17 (2.47%)  62 (9.02%)  22 (3.20%)  3 (0.44%) | 2886 (86.1%)  54 (1.61%)  294 (8.77%)  106 (3.16%)  13 (0.39%) |
| **IMD Quintile**^†^  1 (Most deprived), n(%)  2, n(%)  3, n(%)  4, n(%)  5 (Least deprived), n(%) | 95 (12.3%)  169 (22.0%)  242 (31.4%)  158 (20.5%)  106 (13.8%) | 468 (12.3%)  841 (22.0%)  1199 (31.4%)  785 (20.6%)  526 (13.8%) | 161 (23.4%)  88 (12.8%)  89 (13.0%)  157 (22.9%)  192 (28.0%) | 780 (23.3%)  422 (12.6%)  441 (13.2%)  722 (23.0%)  938 (28.0%) |
| **Weight in kg at baseline**  Mean (SD) Median (IQR) | 87.6 (19.1)  85.4 (73.5, 100.0) | 85.0 (19.2)  82.8 (71.5, 95.8) | 86.8 (19.6)  85.0 (73.0, 98.0) | 86.4 (19.7)  83.6 (72.8, 97.4) |
| **BMI at baseline** ^a^  Mean (SD) Median (IQR) | 31.1 (5.94)  30.1 (26.7, 34.5) | 30.3 (6.1)  29.8 (26.5, 33.8) | 30.3 (6.0)  29.3 (26.1, 33.3) | 30.6 (6.1)  29.6 (26.5, 33.7) |

* Asian’ comprises those reporting Indian, Pakistani, Bangladeshi, Chinese or ‘other Asian’ ethnicity; ‘Black’ comprises those reporting Caribbean, African or ‘other Black’ ethnicity; ‘Mixed’ comprises people with a Mixed ethnic background and ‘Other’ comprises those reporting any other ethnicity.
^†^‘IMD’ – Index of Multiple Deprivation (English, 2015)
^a^ ‘BMI’ – Body Mass Index

**4. Interaction analyses – digital choice**

Table S4 Complete case interaction analyses assessing differential change in weight from baseline to 6 months between the face-to-face cohort and the digital-choice cohort. Subgroup effects are also shown, where relevant

|  | **Change in weight* (n=30854)** | | |
| --- | --- | --- | --- |
|  | **B** | **95% CI** | **p-value** |
| **Sex**  Male (ref) Female  Interaction | -0.829  -1.298  -0.468 | (-1.397, -0.262)  (-1.862, 0.734)  (-1.019, 0.083) | 0.096 |
| **Age at referral**  Interaction | -0.050 | (-0.074, -0.026) | <0.001 |
| **Ethnicity**^‡^  White (ref)  Mixed  Asian  Black  Other  Interaction (Mixed)  Interaction (Asian)  Interaction (Black)  Interaction (Other) | -1.259  0.995  -0.221  1.749  -0.861  0.263  1.038  3.008  0.398 | (-1.765, -0.752)  (-2.797, 0.806)  (-1.188, 0.747)  (0.104, 3.395)  (-4.647, 2.925)  (-1.521, 2.047)  (0.105, 1.971)  (1.395, 4.621)  (-3.379, 4.175) | 0.772  0.029  <0.001  0.837 |
| **IMD^a^**  1 (most deprived) (ref)  2  3  4  5 (least deprived)  Interaction (2)  Interaction (3)  Interaction (4)  Interaction (5) | -0.317  -0.789  -0.568  -1.618  -1.431  0.471  -0.251  -1.301  -1.114 | (-1.057, 0.422)  (-1.660, 0.083)  (-1.439, 0.303)  (-2.311, -0.925)  (-2.094, -0.769)  (-1.423, 0.481)  (-1.237, 0.736)  (-2.139, -0.462)  (-1.953, -0.275) | 0.332  0.618  0.002  0.009 |
| **Weight in kg at baseline**  Interaction | n/a |  |  |

*All models adjust for age at referral, sex, ethnicity (white/mixed/black/Asian/other), IMD quintile, time since baseline (in months) as fixed effects and CCG (Clinical Commissioning Group) nested within STP (Sustainability and Transformation Partnership) as random effects
‡ Asian’ comprises those reporting Indian, Pakistani, Bangladeshi, Chinese or ‘other Asian’ ethnicity; ‘Black’ comprises those reporting Caribbean, African or ‘other Black’ ethnicity; ‘Mixed’ comprises people with a Mixed ethnic background and ‘Other’ comprises those reporting any other ethnicity.
^a^‘IMD’ – Index of Multiple Deprivation (English, 2015)

Older age was associated with greater weight loss in the digital-choice programme in comparison to the face-to-face programme (Table S3). Similar to the comparison of face-to-face and digital-only, evidence of a difference across ethnic groups was seen. White individuals lost more weight on the digital programme than the face-to-face programme and Black individuals lost more weight on the face-to-face programme than the digital programme. The interaction effect between White and Black ethnic groups was 3.008 (95% CI: 1.395, 4.621) and the interaction effect between White and Black individuals was 1.038 (95% CI: 0.105, 1.971) suggesting that the difference in weight loss between White and Black individuals and White and Asian individuals was statistically significantly larger in the digital programme than the face-to-face programme. Again, results should be viewed with caution due to the small number of Black and Asian individuals in the digital cohort.

On average, weight loss was greater in the digital-choice group compared to the face-to-face group in all deprivation quintiles: this difference was smaller in magnitude and not statistically significant for quintiles 1, 2 and 3 yet larger in magnitude and statistically significant in quintiles 4 and 5: individuals in quintile 4, on average, lost 1.618kg (95% CI: 0.925, 2.311) more weight on the digital programme compared to the face-to-face programme and individuals in quintile 5, on average, lost 1.431kg (95% CI: 0.769, 2.094) more weight on the digital programme compared to the face-to-face programme. The interaction effects comparing quintiles 4 and 5 with quintile 1 were statistically significant and suggested the difference in weight loss between the least deprived and the most deprived was larger in the digital programme than the face-to-face programme.

**5. Additional analyses**

***5.1 Analyses using regression adjustment to account for confounding***

We re-ran the main analysis using regression adjustment to account for confounding instead of matching. The confounding variables adjusted for were sex, age at referral, deprivation and ethnicity. Again, mixed effects linear regression was used additionally adjusting for CCG nested within STP as random effects. Results are shown in Tables S4 and S5.

Table S5 Regression analyses comparing change in weight from baseline to 6 months and 12 months between the face-to-face cohort and the digital-only cohort using regression adjustment to account for confounding

|  | **n** | **B*** | **95% CI** | **p-value** |
| --- | --- | --- | --- | --- |
| **Weight in kg at 6 months**  Unadjusted  Adjusted^†^ | 33769  31064 | -0.204  -0.136 | (-0.449, 0.041)  (-0.620, 0.347) | 0.102  0.581 |
| **Weight in kg 12 months**  Unadjusted  Adjusted^†^ | 24276  22278 | 0.142  -0.423 | (-0.177, 0.462)  (-0.984, 0.138) | 0.382  0.140 |

*Coefficient quantifies the difference in mean change between the face-to-face and digital cohort, using the face-to-face cohort as the reference group
† Model adjusts for age at referral, sex, ethnicity (white/mixed/black/Asian/other), IMD (Index of Multiple Deprivation) quintile, time since baseline (in months) as fixed effects and CCG (Clinical Commissioning Group) nested within STP (Sustainability and Transformation Partnership) as random effects

Table S6 Regression analyses comparing change in weight from baseline to 6 months and 12 months between the face-to-face cohort and the digital-choice cohort using regression adjustment to account for confounding

|  | **n** | **B*** | **95% CI** | **p-value** |
| --- | --- | --- | --- | --- |
| **Weight in kg at 6 months**  Unadjusted  Adjusted^†^ | 33574  30854 | -0.943  -1.067 | (-1.214, -0.671)  (-1.566, -0.568) | <0.001  <0.001 |
| **Weight in kg at 12 months**  Unadjusted  Adjusted^†^ | 24223  22203 | -0.347  -1.001 | (-0.677, -0.017)  (-1.522, -0.497) | 0.039  <0.001 |

*Coefficient quantifies the difference in mean change between the face-to-face and digital cohort, using the face-to-face cohort as the reference group
† Model adjusts for age at referral, sex, ethnicity (white/mixed/black/Asian/other), IMD (Index of Multiple Deprivation) quintile, time since baseline (in months) as fixed effects and CCG (Clinical Commissioning Group) nested within STP (Sustainability and Transformation Partnership) as random effects

Overall, the results obtained using matching and regression adjustment are very similar and lead to the same conclusions. At both 6 and 12 months, weight loss in the digital-only and face-to-face cohorts was similar and the upper limits of the 95% confidence intervals for the adjusted mean differences were below the pre-specified non-inferiority limits, demonstrating non-inferiority.

At both 6 and 12 months, weight loss in the digital-choice group was approximately 1kg greater than that in the face-to-face cohort, on average, and this was statistically significant. It follows that non-inferiority was demonstrated.

***5.2 Analyses using multiple imputation to account for missing data***

The main analysis was a complete case analysis whereby only observations with complete data were used. The analysis was additionally run using multiple imputation, imputing missing baseline data in both cohorts, as well as missing outcome data in the face-to-face cohort for individuals known to be participating in the programme at the time the measurement was expected.

In the digital cohort, missing baseline information was imputed for individuals who had an observed baseline and 6/12m weight value. Regression imputation was used to impute missing values in variables with a small amount of missing data: 6 missing sex values, 1 missing age value and 3 missing IMD quintile values. Multiple imputation by chained equations was used to impute 136 missing ethnicity values.

In the face-to-face cohort, as well as imputing missing baseline information, multiple imputation was used to impute missing outcome values for individuals who were still attending the programme at the time of the measurement in the face-to-face cohort.

1. 6m weight analysis. In the complete case adjusted regression analysis, there were 30090 individuals in the face-to-face cohort. Regression imputation was used to impute missing values in variables with a small amount of missing data: 71 missing sex values and 68 missing IMD quintile values (there were no missing age values). Multiple imputation by chained equations was used to impute 2745 ethnicity values, 1612 baseline weight values and 752 6m weight values, resulting in a sample of 34800.
2. 12m weight analysis. In the complete case adjusted regression analysis, there were 21503 individuals in the face-to-face cohort. Regression imputation was used to impute missing values in variables with a small amount of missing data: 71 missing sex values and 68 missing IMD quintile values (there were no missing age values). Multiple imputation by chained equations was used to impute 2021 ethnicity values, 1182 baseline weight values and 619 12m weight values, resulting in a sample of 25068.

Table S6 and S7 show the results of the regression analyses comparing change in weight from baseline to 6 and 12 months between the face-to-face cohort and the digital-only and digital-choice cohorts respectively. Differences in sample size between the unadjusted and adjusted analyses are due to missing CCG or STP values as these were not imputed.

Table S7 Regression analyses using multiple imputation comparing change in weight from baseline to 6 months and 12 months between the face-to-face cohort and the digital-only cohort

|  | **n** | **B*** | **95% CI** | **p-value** |
| --- | --- | --- | --- | --- |
| **Weight in kg at 6 months**  Unadjusted  Adjusted^†^ | 35825  35802 | -0.200  -0.181 | (-0.445, 0.044)  (-0.642, 0.280) | 0.108  0.442 |
| **Weight in kg at 12 months**  Unadjusted  Adjusted^†^ | 25886  25862 | 0.142  -0.243 | (-0.177, 0.460)  (-0.785, 0.299) | 0.383  0.380 |

*Coefficient quantifies the difference in mean change between the face-to-face and digital cohort, using the face-to-face cohort as the reference group
† Model adjusts for age at referral, sex, ethnicity (white/mixed/black/Asian/other), IMD (Index of Multiple Deprivation) quintile, time since baseline (in months) as fixed effects and CCG (Clinical Commissioning Group) nested within STP (Sustainability and Transformation Partnership) as random effects

Table S8 Regression analyses using multiple imputation comparing change in weight from baseline to 6 months and 12 months between the face-to-face cohort and the digital-choice cohort

|  | **n** | **B*** | **95% CI** | **p-value** |
| --- | --- | --- | --- | --- |
| **Weight in kg at 6 months**  Unadjusted  Adjusted^†^ | 35630  35625 | -0.939  -0.935 | (-1.209, -0.668)  (-1.403, -0.468) | <0.001  <0.001 |
| **Weight in kg at 12 months**  Unadjusted  Adjusted^†^ | 25833  25828 | -0.348  -0.720 | (-0.676, -0.019)  (-1.214, -0.225) | 0.038  0.004 |

*Coefficient quantifies the difference in mean change between the face-to-face and digital cohort, using the face-to-face cohort as the reference group
† Model adjusts for age at referral, sex, ethnicity (white/mixed/black/Asian/other), IMD (Index of Multiple Deprivation) quintile, time since baseline (in months) as fixed effects and CCG (Clinical Commissioning Group) nested within STP (Sustainability and Transformation Partnership) as random effects

In comparison to the results from the complete case analysis, the point estimates are similar but the results are more precise as the sample size has been substantially increased with the multiple imputation.

Tables S8 and S9 show the results of the interaction analyses comparing the difference in change in weight across the baseline variables between the face-to-face and digital-only and digital-choice cohorts respectively.

Table S9 Interaction analyses using multiple imputation assessing differential change in weight from baseline to 6 months between the face-to-face cohort and the digital-only cohort. Subgroup effects are shown, where relevant

|  | **Change in weight* (n=35802)** | | |
| --- | --- | --- | --- |
|  | **B** | **95% CI** | **p-value** |
| **Sex**  Male (ref) Female  Interaction | 0.394  -0.634  -1.029 | (-0.110, 0.899) (-1.108, -0.161)  (-1.514, -0.543) | <0.001 |
| **Age at referral**  Interaction | 0.006 | (-0.016, 0.027) | 0.622 |
| **Ethnicity**^‡^  White (ref)  Mixed  Asian  Black  Other  Interaction (Mixed)  Interaction (Asian)  Interaction (Black)  Interaction (Other) | -0.464  -0.190  0.534  1.080  -2.820  0.275  0.998  1.544  -2.356 | (-0.931, 0.002)  (-2.327, 1.948)  (-0.285, 1.353) (-0.081, 2.241) (-7.982, 2.342)  (-1.867, 2.416)  (0.122, 1.874)  (0.335, 2.753)  (-7.523, 2.812) | 0.801  0.026  0.012  0.372 |
| **IMD^a^**  1 (most deprived) (ref)  2  3  4  5 (least deprived)  Interaction (2)  Interaction (3)  Interaction (4)  Interaction (5) | -0.686  0.417  -0.246  -0.530  -0.001  1.102  0.439  0.155  0.685 | (-1.422, 0.051)  (-0.200, 1.034)  (-0.831, 0.339)  (-1.188, 0.127)  (-0.771, 0.769)  (0.270, 1.934)  (-0.388, 1.267)  (-0.725, 1.035)  (-0.291, 1.661) | 0.009  0.298  0.729  0.169 |
| **Weight at baseline**  Interaction | n/a |  |  |

*All models adjust for age at referral, sex, ethnicity (white/mixed/black/Asian/other), IMD quintile, time since baseline (in months) as fixed effects and CCG nested within STP as random effects
‡ Asian’ comprises those reporting Indian, Pakistani, Bangladeshi, Chinese or ‘other Asian’ ethnicity; ‘Black’ comprises those reporting Caribbean, African or ‘other Black’ ethnicity; ‘Mixed’ comprises people with a Mixed ethnic background and ‘Other’ comprises those reporting any other ethnicity.
^a^‘IMD’ – Index of Multiple Deprivation (English, 2015)

Table S10 Interaction analyses using multiple imputation assessing differential change in weight from baseline to 6 months between the face-to-face cohort and the digital-choice cohort. Subgroup effects are shown, where relevant

|  | **Change in weight* (n=35802)** | | |
| --- | --- | --- | --- |
|  | **B** | **95% CI** | **p-value** |
| **Sex**  Male (ref) Female  Interaction | -0.719  -1.105  -0.386 | (-1.250, -0.189) (-1.637, -0.573)  (-0.914, 0.143) | 0.153 |
| **Age at referral**  Interaction | -0.051 | (-0.074, -0.027) | <0.001 |
| **Ethnicity**^‡^  White (ref)  Mixed  Asian  Black  Other  Interaction (Mixed)  Interaction (Asian)  Interaction (Black)  Interaction (Other) | -1.124  -0.858  -0.009  1.608  -0.835  0.266  1.115  2.732  0.289 | (-1.601, -0.648)  (-2.613, 0.898) (-0.929, 0.910) (-0.183, 3.340)  (-4.143, 2.473)  (-1.484, 2.017)  (0.217, 2.012) (0.946, 4.519)  (-3.020, 3.598) | 0.765  0.015  0.003  0.864 |
| **IMD^a^**  1 (most deprived) (ref)  2  3  4  5 (least deprived)  Interaction (2)  Interaction (3)  Interaction (4)  Interaction (5) | -0.195  -0.692  -0.267  -1.500  -1.379  -0.497  -0.071  -1.305  -1.184 | (-0.879, 0.488)  (-1.501, 0.116)  (-1.086, 0.553)  (-2.167, -0.833)  (-2.018, 0.740)  (-1.385, 0.390)  (-1.000, 0.857) (-2.109, -0.500) (-1.986, -0.382) | 0.272  0.880  0.001  0.004 |
| **Weight at baseline**  Interaction | n/a |  |  |

*All models adjust for age at referral, sex, ethnicity (white/mixed/black/Asian/other), IMD quintile, time since baseline (in months) as fixed effects and CCG (Clinical Commissioning Group) nested within STP (Sustainability and Transformation Partnership) as random effects
‡ Asian’ comprises those reporting Indian, Pakistani, Bangladeshi, Chinese or ‘other Asian’ ethnicity; ‘Black’ comprises those reporting Caribbean, African or ‘other Black’ ethnicity; ‘Mixed’ comprises people with a Mixed ethnic background and ‘Other’ comprises those reporting any other ethnicity.
^a^‘IMD’ – Index of Multiple Deprivation (English, 2015)

All interactions effects were very similar to those from the complete case analysis and the conclusions are the same.

***5.3 Analyses changing the time of the baseline measurement in the face-to-face cohort***

The main analysis used the first intervention session as baseline in the face-to-face cohort as this is when participants first received programme content. A sensitivity analysis was run redefining baseline in face-to-face group as the initial assessment as it is plausible that some individuals may have started to make lifestyle changes after this first interaction, prior to the first session attended. Registration was kept as the baseline time point in the digital cohort.

Table S10 shows the raw changes in weight from baseline to 6 and 12 months in the face-to-face cohort and the two digital cohorts. In both the face-to-face and digital-choice cohorts, a reduction in weight, on average, was observed at both 6 and 12 months. In the face-to-face cohort, change from initial assessment to 6 and 12 months in weight was similar to change from first intervention session shown in the main analysis.

Table S11 Summary of weight outcome measures of participants in the face-to-face cohort and digital-only cohort where initial assessment was the baseline time point in the face-to-face cohort

|  | **Face-to-face** | **Digital only** | **Digital choice** |
| --- | --- | --- | --- |
| **Weight in kg at 6 months**  n Baseline; Mean (SD)  6m; Mean (SD) Change; Mean (95% CI) | 32476  83.52 (18.28)  81.14 (18.01)  -2.38 (-2.42, -2.34) | 1025  87.43 (19.15)  84.37 (18.65)  -3.05 (-3.38, -2.73) | 830  86.98 (19.04)  83.18 (18.85)  -3.79 (-4.16, -3.43) |
| **Weight in kg at 12 months**  n Baseline; Mean (SD)  12m; Mean (SD) Change; Mean (95% CI) | 24799  83.05 (17.99)  80.21 (17.77)  -2.85 (-2.90, -2.80) | 818  87.42 (19.07)  84.52 (18.89)  -2.90 (-3.31, -2.48) | 765  86.92 (19.49)  83.53 (19.50)  -3.39 (-3.86, -2.91) |

Tables S11 and S12 show the output from the linear mixed model analyses comparing change in weight from baseline to 6 and 12 months between the face-to-face and digital-only and digital-choice cohorts respectively where initial assessment was used as the baseline time point in the face-to-face cohort. Overall, some point estimates are different to that in the main analysis but changing the timing of baseline in the face-to-face cohort has not impacted the overall conclusions.

Table S12 Regression analyses comparing change in weight from baseline to 6 months and 12 months between the face-to-face cohort and the digital-only cohort where initial assessment was the baseline time point in the face-to-face cohort

|  | **n** | **B*** | **95% CI** | **p-value** |
| --- | --- | --- | --- | --- |
| **Weight in kg at 6 months**  Unadjusted  Adjusted^†^ | 33501  30859 | -0.434  -0.381 | (-0.689, -0.178)  (-0.885, 0.122) | 0.001  0.138 |
| **Weight in kg at 12 months**  Unadjusted  Adjusted^†^ | 25617  23610 | 0.175  -0.241 | (-0.158, 0.509)  (-0.820, 0.337) | 0.303  0.414 |

*Coefficient quantifies the difference in mean change between the face-to-face and digital cohort, using the face-to-face cohort as the reference group
† Model adjusts for age at referral, sex, ethnicity (white/mixed/black/Asian/other), IMD (Index of Multiple Deprivation) quintile, time since baseline (in months) as fixed effects and CCG (Clinical Commissioning Group) nested within STP (Sustainability and Transformation Partnership) as random effects

Table S13 Regression analyses comparing change in weight from baseline to 6 months and 12 months between the face-to-face cohort and the digital-choice cohort where initial assessment was the baseline time point in the face-to-face cohort

|  | **n** | **B**^‡^ | **95% CI** | **p-value** |
| --- | --- | --- | --- | --- |
| **Weight in kg at 6 months**  Unadjusted  Adjusted^†^ | 33306  30649 | -1.172  -1.373 | (-1.456, -0.889)  (-1.881, -0.866) | <0.001  <0.001 |
| **Weight in kg at 12 months**  Unadjusted  Adjusted^†^ | 25564  23535 | -0.314  -0.701 | (-0.658, 0.031)  (-1.213, -0.171) | 0.074  0.010 |

*Coefficient quantifies the difference in mean change between the face-to-face and digital cohort, using the face-to-face cohort as the reference group
† Model adjusts for age at referral, sex, ethnicity (white/mixed/black/Asian/other), IMD (Index of Multiple Deprivation) quintile, time since baseline (in months) as fixed effects and CCG (Clinical Commissioning Group) nested within STP (Sustainability and Transformation Partnership) as random effects
